# A novel code generator for graphical user interfaces

**DOI:** 10.1038/s41598-023-46500-6

**Published:** 2023-11-21

**Authors:** Bo Cai, Jian Luo, Zhen Feng

**Affiliations:** 1 School of Cyber Science and Engineering, Wuhan University, Wuhan, 430072 China; 2 Key Laboratory of Aerospace Information Security and Trusted Computing, Ministry of Education, Wuhan, 430072 China

**Keywords:** Computer science, Information technology, Software

## Abstract

Graphical user interfaces (GUIs) are widely used in human–computer interaction, providing a convenient interface for operation. Automating the conversion of GUI design images into source code can significantly reduce the coding workload for front-end developers. Detecting elements in GUI images is a key challenge in achieving automatic GUI code generation and is crucial for tasks such as GUI automation and testing. However, current state-of-the-art methods do not fully consider the unique characteristics of GUI images and elements, and they lack the required high localization accuracy, resulting in low detection accuracy for GUI element boxes. In this paper, we propose GUICG, an automatic GUI code generator that combines deep neural networks with image processing techniques to efficiently detect GUI elements from GUI images and generate front-end code. We empirically investigate various deep learning approaches and image processing methods for GUI component detection. Based on a comprehensive understanding of their performance and characteristics, we design GUICG by fusing image processing with a deep learning-based target detection model, achieving state-of-the-art performance. GUICG outperforms existing methods in accuracy and F1 score for component detection tasks, while producing human-readable code with a logical structure. Furthermore, we conduct an ablation study to quantitatively assess the impact of each key element in GUICG.

## Introduction

Graphical user interface (GUI) is a very convenient way of human–computer interaction^1^, software relies on simple user interface (UI) and intuitive user experience to attract users. The user interface of the software requires the designer to design the GUI and then convert it to the front-end code through the front-end engineer. This process often consumes a lot of time and energy^2^. In addition, the implementation codes of the graphical user interface of the software on different operating systems are different, which leads to the need for code conversion by front-end engineers. Automated code generation can significantly reduce the workload of front-end developers, allowing them to focus on other critical aspects of software development. Therefore, automatically converting UI design into executable code will greatly improve the developer’s work efficiency.

The method proposed by Chen et al. combines traditional image processing methods for non-text area detection with deep learning models for area classification and GUI text detection^3^. For GUI text detection, they use the pre-trained scene text detector EAST. For non-text GUI component detection, a two-stage design is adopted, that is, region detection and region classification are performed sequentially. For region detection, they developed an image processing method with a top-down coarse strategy and a set of GUI-specific image processing algorithms. For region classification, a pretrained ResNet50 image classifier fine-tuned for GUI component images is used. Although Chen et al. tested a variety of methods, the methods they used were all methods from a long time ago .

At present, the basic process of GUI code generation based on computer vision is to use computer vision technology to process GUI images, and then convert them into front-end codes. According to the different computer vision technologies adopted, such methods can be divided into GUI code generation methods based on deep learning object detection methods and non-deep learning GUI code generation methods based on traditional image processing^4^. Image processing-based techniques rely on aggregation heuristics generated from image features and expert knowledge, while deep learning object detection-based techniques use CNNs to obtain image features and their aggregation rules from large amounts of GUI image data. GUI graphics processing tasks are different from computer vision tasks in natural scenes, and graphics have distinct characteristics. Therefore, we explored various models of deep neural networks and investigated whether their structures and methods are useful for our research, optimized in conjunction with image processing algorithms.

In this paper, we propose a new method for GUI code generation based on component detection, called GUICG. In the GUI component detection phase, we introduce several improvements to enhance the performance of the image processing algorithm and the deep learning target detection algorithm for GUI component features. Additionally, we develop a fusion design that combines image processing and target detection, addressing the high localization accuracy required for GUI component detection. Moreover, we consider the problem of text on GUI components and restore it using text detection and recognition techniques.

To accomplish our task, we divide it into two steps. The first step involves detecting and classifying GUI components into various types (e.g., buttons, images) and representing them as specific GUI objects using a fusion of target detection models and deep learning-based image processing. In the second step, we generate the corresponding code using a code generator. We improve both the deep learning target detection and image processing methods to meet the requirements of GUI component detection, taking into account the unique features of GUI components, such as shape, texture, boundaries, and layout. For GUI text detection, we utilize the FOTS model^5^, an established end-to-end scene text detection and recognition model, to detect and recognize text components.

Our proposed fusion-based approach for GUI component detection achieves a significant improvement in performance compared to current state-of-the-art methods. In an evaluation of over 10,000 GUI images, we surpass existing computer vision-based and image processing-based algorithms, achieving an F1 score of 54.32% for all GUI components, which is 2% higher than the current state-of-the-art approaches.

The contributions of this paper are as follows:

- We conduct an empirical study to evaluate target detection methods and image processing methods for GUI component detection, providing insights into their effectiveness and limitations, which serve as the foundation for our proposed method.
- We propose GUICG, an integrated generative model that combines deep neural networks and image processing techniques to overcome the challenges in translating GUI images to code.
- We extensively evaluate GUICG using mainstream datasets and compare it with current state-of-the-art methods in the field. Additionally, we conduct ablation experiments to assess the impact and improvements of each change in the network. The experimental results demonstrate that GUICG outperforms existing methods across multiple evaluation metrics, effectively combining the advantages of image processing and target detection methods.

Overall, our proposed GUICG method significantly improves the performance of GUI component detection in GUI code generation, providing more accurate code generation and a reasonable code structure.

## Related work

Automatically generating code from GUI designs using deep learning techniques is a relatively new area of research. This process involves the problem of machine understanding the images of the design and extracting logical information from them, so this task can be seen as a computer vision problem. Therefore, we present the related work in this section in three parts: GUI object detection, GUI text recognition and GUI code generation, respectively.

### GUI component detection

The first step in the GUI image generation code task is GUI component detection, which is actually similar to the object detection problem of computer vision, which is used to detect elements in the GUI^6–8^ use traditional image processing methods to detect GUI components^9–12^, detect GUI components based on deep learning methods. Component detection based on traditional target detection methods requires manual feature extraction. Candidate boxes are obtained through sliding windows, and then traditional classifiers are used to determine target areas. The entire training process is divided into multiple steps. For deep learning-based methods, end-to-end object detection can be performed without defining features, usually based on convolutional neural networks (CNN). The target detection method based on deep learning can be divided into two types: One-stage^13–15^ and Two-stage^10,16,17^, and there is also a RefineDet^18^ algorithm that inherits the advantages of these two types of methods.

### GUI text recognition

The graphic recognition network recognizes the segmented text area image block into text content. CRNN^19^ is currently a more popular graphic recognition network, which can recognize relatively long and variable text sequences. The feature extraction layer includes CNN and BLSTM, which can carry out end-to-end joint training. It uses BLSTM and connectionist temporal classification (CTC) network to learn the context relationship between character images, thereby effectively improving the accuracy of image recognition. RARE^20^ network is relatively effective in recognizing distorted image text. In the model inference process, the input image passes through the spatial transformation network to obtain the corrected image; then, the corrected image enters the sequence recognition network, and finally the text prediction result is obtained. ESIR^21^ end-to-end scene text recognition consists of two parts, one is an iterative text correction network, and the other is a sequence recognition network.

### GUI code generation

pix2code^22^ uses screenshots of the application user interface and the corresponding DSL as training data at the same time. The code generation process is complicated and the workload is heavy. The model uses the LSTM network of the pix2code model, which needs to be retrained for each different platform, and this repeated training increases the total time of code generation. Existing methods (e.g., Pix2code) can handle simple datasets well but have difficulty handling complex datasets that require hundreds of code tokens.Based on this problem, a front-end code generation method based on multiple heads of attention is proposed, which uses a special technique known as multiple heads of attention to analyze the feature vectors of the GUI screenshots, generate the code tokens, and link the analyzing and generating processes^23^.

Sketch2Code^24^ is a model for generating code from design sketches. It consists of a convolutional neural network built on top of the RetinaNet^15^ object detection architecture, which is a fast and time-efficient single-stage detection method. However, because it uses sketch images, differences in shooting effects (such as light changes) will have a greater impact on the inference efficiency of the model. The REDRAW^6^ data collection and training process can be performed iteratively and fully automatically over time, helping to reduce the burden on developers. It divides the task into three parts, but the detection and classification can be combined into one, so as to improve the efficiency of the model operation. However, REDRAW is currently limited to detecting and combining a few specific sets of style details from GUI images^25^ integrates CNN, RNN encoder and RNN decoder in a unified framework. The RNN encoder encodes the spatial layout information of these image features into a summary vector, and then the RNN decoder uses the summary vector to generate a GUI framework for token sequence representation.

Recently some new researches have improved the experimental methodology and evaluation metrics, propose a new modeling architecture to improve the framework of pix2code, which also automatically generates platform-specific code as an input for a given GUI screenshot, and in order to overcome the limitations of BLEU in domain-specific language (DSL) token evaluation, we introduce an improved BLEU score (MBLEU)^26^. Our work in developing a novel code generator for Graphical User Interfaces is guided by the need to address contemporary challenges in software engineering. These challenges include the proliferation of unknown malware in the digital landscape^27^ and security vulnerabilities arising from artificial intelligence algorithms^28^. We draw inspiration from the insights offered by Qiu^29^. Their work emphasizes the importance of knowledge extraction in the smart city landscape. They introduce innovative methods, such as Semantic Graph-Based Concept Extraction (SGCCE), which harnesses semantic information for enhanced concept extraction. This resonates with our own objective of creating an intelligent GUI code generator, rooted in the effective utilization of information.

The above methods are designed without considering the unique features of GUI images and GUI elements separately, and without considering the problem of high localization accuracy that is necessary for GUI code generation tasks. We fully consider these issues and propose a novel GUI code generation model. Integrating image processing and deep learning object detection in the detection stage of GUI elements brings significant improvements in accuracy, recall and localization precision by fusing the advantages of both methods.

## Analysis of component detection model in code generation of GUI

In this section, we will study the effectiveness of general object detection and image processing methods applied to GUI component detection. Firstly, the unique characteristics of GUI images, GUI components and unique requirements of GUI component detection tasks are summarized, and then the problem of general object detection in the face of these unique characteristics of GUI is proposed. Further, this section will design an exploratory experiment to explore the effectiveness and limitations of several typical GUI component detection methods, and provide effective design ideas in the Methodology section.

### GUI component detection problem analysis

The components in the GUI image are different from the object detection in the natural scene. The distance between the components on the GUI image is very close, and their arrangement is very dense^4^. A GUI image will have many differences, components are mixed. Analyzing some GUI images in the data set, it can be seen from the data that GUI components have the following characteristics: There is a large similarity between components, such as TextView and EditView, both of which are text boxes, which are highly similar in GUI images; the same Class components have different forms, such as: the components in the Button class have different forms, and the forms of the same component are quite different; at the same time, the GUI component detection task requires high component bounding box accuracy. Based on the characteristics of GUI images and components, we need to explore what methods are effective for GUI component detection.

Image processing-based GUI component detection methods usually rely on edge or silhouette aggregation^6,7^. The Canny edge and contour maps used in its method are the visual features of objects in natural scenes, which aim to capture the fine-grained texture details of objects. However, since GUI images may contain images of natural scenes, applying these fine-grained features to GUI components can easily lead to wrong detection results. Deep learning-based methods^3,9,12,30,31^ can effectively learn the characteristics of GUI components for correct detection?

Faced with the intra-class differences, component density, and component similarity of components in GUI images, can this bounding box regression method meet the high accuracy requirements of GUI component detection?

This article defines GUI text components as components such as TextView that only contain text, and its function is to display text. Although components such as Button have text but have a click function, they do not belong to this category. Most methods to detect text components use OCR tools (such as Tesseract^32^). OCR tool or scene text model which is more effective in detecting GUI text?

To sum up, the three research questions of GUI component detection task analysis in this section are as follows:

- Which method is more suitable for feature detection of GUI components?
- How well do different types of methods perform in predicting bounding boxes of GUI components?
- How should the detection of GUI text components and the recognition of text in components be handled?

### Exploratory experimental analysis of GUI component detection

In order to answer the above questions, this paper first conducts analytical experiments to explore the performance of various technologies in the GUI component detection task. The experiments will use image processing and deep learning methods for GUI component detection respectively. This study is conducted on the Rico dataset^33^. This research involves a systematic comparison of two computer vision methods, representative methods based on image processing include REMAUI^7^, UIED^8^ and UI2CODE^34^; three different types of mature deep learning objectives Detection methods include two anchor box-based methods Faster RCNN^16^ (two-stage model) and YOLOv4^9^ (single-stage model) and a single-stage anchor-free model FCOS^12^. For GUI text detection, the performance of the OCR tool Tesseract^32^, the scene text detector EAST^35^, and the end-to-end text detection and recognition model FOTS^5^ are compared, and the separate detection of text and non-text GUI components is compared The performance difference with unified detection.

#### Dateset

This paper uses the Rico dataset^33^ as the dataset for the exploratory experiments in this section. The information of the dataset is shown in Table 1. The experiment can deal with 15 commonly used components in the Android application interface. We randomly divide it into three parts, of which the proportions are 80%, 10%, 10% for training, validation and testing. It is worth noting that all GUIs of the same application will only appear in the same split to avoid the problem of “seen samples” in training, validation and testing, and a 5-fold crossover is performed in all experiments verify.

**Table 1:** The statistics of Rico dataset.

Items	Quantity
Application categories	27
Android mobile applications	9300
GUI images	66,261
GUI elements	986,731
Non-text elements	478,404
Text elements	508,327

#### Model training settings

This experiment trains each model for 400 iterations with a batch size of 16 or 32 depending on the method, and uses the Adam^36^ optimizer. Faster RCNN uses ResNet-101^35^ as the backbone. YOLOv4 uses CSPDarknet-53^9^ as the backbone. FCOS^35^ uses ResNeXt-64x4d-101-FPN as the backbone network. For UI2CODE, REMAUI and UIED, this article uses their best settings. In all experiments in this section, non-maximum suppression (Non-Maximum Suppression, NMS) was performed to remove highly repetitive predictions. The data listed in this experiment table are the data of the best performance of each model on the validation dataset.

#### Evaluation

This paper uses Precision, Recall and F1-score to evaluate the performance of all methods. For the GUI component detection task in this section, the following definitions are made. A detected bounding box is considered positive if its highest IoU with all ground-truth bounding boxes in the input GUI image is higher than a predetermined IoU threshold. The true positive case (True Positive, TP) is that the detected bounding box matches the real bounding box; the false positive case (FP) is that the detected box does not match any real bounding box; the false negative case (FN) for ground-truth bounding boxes that do not match any detected boxes. The precision (Prec) calculation formula is as follows:

1 $$\begin{aligned} Prec = \frac{TP}{TP+FP} \end{aligned}$$

The formula for calculating the recall rate (Rec) is as follows:

2 $$\begin{aligned} Rec = \frac{TP}{TP+FN} \end{aligned}$$

The formula for calculating the F1 score is as follows:

3 $$\begin{aligned} F1 = \frac{2\times Prec \times Rec}{Prec + Rec} \end{aligned}$$

The calculation method of IoU is to divide the intersection area of the two predicted bounding boxes *A* and *B* by the union area of the two bounding boxes, where *I* represents the intersection area of the two bounding boxes, the formula is as follows:

4 $$\begin{aligned} IoU = \frac{I}{A+B-I} \end{aligned}$$

**Figure 1:**
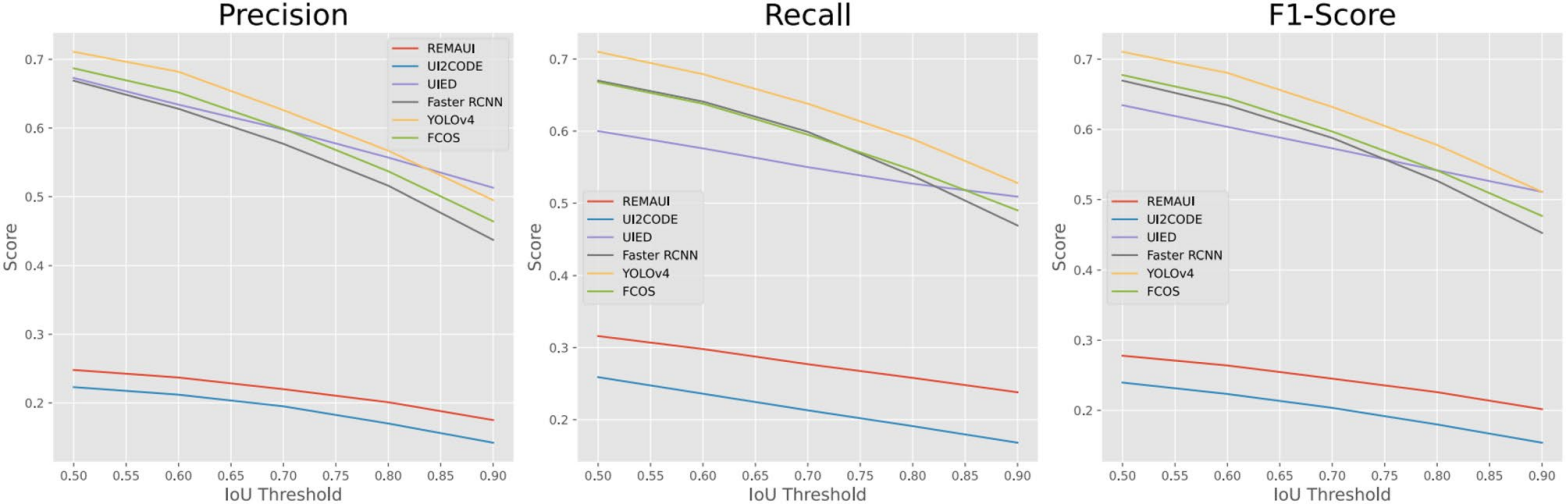
Performance of precision, recall and F1 score of six representative methods(REMAUI, UI2CODE, UIED, Faster RCNN, YOLOv4, FCOS) used in performance analysis of component detection model under different IoU thresholds.

### Analysis of GUI component detection experiment results

This section will show the performance of six methods (REMAUI, UI2CODE, UIED, Faster RCNN, YOLOv4, FCOS) applied to GUI component detection and analyze them to explore the performance of various types of methods on GUI detection tasks features. Firstly, this paper wants to explore the impact of different IoU thresholds on model performance, keeping all settings except the IoU threshold unchanged, the performance of the six methods under different IoU thresholds is shown in Fig. 1. In this section, every 0.05 of 0.5–0.9 is selected as the experimental IoU threshold setting. When the IoU threshold increases from 0.5 to 0.9, the F1 scores of all deep learning models decrease significantly, and Faster-RCNN, YOLOv4 and FCOS decrease by 32.4%, 28.1% and 29.6%. The deep learning model is able to detect more components, but its bounding box is not precise enough, i.e., the deep learning method has a high recall rate, but the bounding box localization is not accurate enough. In contrast, the F1 scores of REMAUI and UI2CODE did not decrease significantly with the increase of the IoU threshold like the deep learning models, but their F1 scores were much lower than those of the deep learning models. This shows that component regions detected by these image processing-based methods are partly noisy, but when they do localize real components, the detected bounding boxes are quite accurate. Due to its advanced design, UIED even performs better than some deep learning methods in terms of accuracy, and its F1 score declines smoothly and is much higher than other image processing-based methods.

**Table 2:** GUI component detection performance of typical methods.

Method	Precision	Recall	F1-score
REMAUI	17.53	23.81	20.19
UI2CODE	14.22	16.86	15.43
UIED	51.34	50.90	51.12
Faster RCNN	43.72	46.96	45.28
YOLOv4	49.57	52.81	51.14
FCOS	46.45	49.02	47.70

When the IoU between the bounding box detected by the GUI component and the real GUI component box is less than 0.9, the bounding box is likely to miss a part of the component, and due to the close arrangement of the GUI components, it is likely to include some adjacent components, so have a serious impact on the next task. Therefore, this paper uses IoU > 0.9 as an acceptable bounding box prediction threshold. Table 2 shows the overall performance of the six methods for detecting GUI components with a threshold of IoU > 0.9. Among them, UI2CODE performed the worst, with all indicators below 17 %.

**Table 3:** The speed of different models processing the same GUI image.

Method	Model	Time (s)
Image processing	REMAUI	5.3
	UI2CODE	1.2
	UIED	4.8
Deep learning	Faster RCNN	0.38
	YOLOv4	0.12
	FCOS	0.28

Meanwhile, this section conducts experiments to explore the processing speed of these representative methods. As shown in Table 3, the deep learning target detection method is significantly faster than the image processing-based method, because the image processing method contains many operations directly on the image, which is much slower than the deep learning method. However, the two-stage method uses separate processing for candidate region proposal and prediction, which leads to a large amount of time for the two-stage method. Therefore, the single-stage method in the target detection method is significantly faster than the two-stage method. Because the UIED method is based on image processing and combines text detection methods, its speed is slow, and REMAUI is the slowest.

### Performance analysis of text component detection and recognition

In this section, experiments are designed to verify this issue, and this section explores whether only one model can be used for reliable text component and non-text component detection at the same time. Therefore, the experiment in this section trained YOLOv4, Faster RCNN and FCOS models on the non-text component training set and the mixed training set of non-text and text components to explore the possibility of a single model to simultaneously detect text and non-text components.

The performance results of different training schemes are shown in Table 4. When both text and non-text GUI components are detected, YOLOv4 is still the best in detecting non-text components. However, it is comparable to when only non-text components are trained. Compared with that, the performance of the three models to detect non-text components has a certain decline. This shows that mixing text and non-text components for training will interfere with the model’s ability to learn the characteristics of non-text components. FCOS has the best overall performance in this experiment, because FCOS is an anchor-free model that can flexibly handle text components that are very different from non-text components, so its performance in detecting text and non-text components is similar. However, there are gaps between words and lines in the text component, and FCOS often regards a word or a sentence as a whole component, which will affect the performance of FCOS in the text component.

**Table 4:** The performance results of different training schemes.

Method	Training scheme	Precision	Recall	F1-score
Faster RCNN	Non-text elements	43.72	46.96	45.28
	Non-text elements (mixed training)	36.53	44.37	40.07
	Text elements (mixed training)	26.45	23.93	25.13
	All elements (mixed training)	35.33	37.42	36.34
FCOS	Non-text elements	46.45	49.02	47.70
	Non-text elements (mixed training)	32.71	45.39	38.02
	Text elements (mixed training)	42.64	39.7	41.12
	All elements (mixed training)	41.46	48.71	44.79
YOLOv4	Non-text elements	49.57	52.81	51.14
	Non-text elements (mixed training)	38.99	48.23	43.12
	Text elements (mixed training)	32.92	31.43	32.16
	All elements (mixed training)	39.77	48.83	43.84

According to the above experiments, it is not feasible to detect both text and non-text GUI components in one model, so it is necessary to study a method suitable for GUI text component detection. Current works such as REMAUI and UI2CODE simply use OCR tools such as Tesseract, while UIED employs the scene text detector EAST. In the experiment, the pre-trained model is directly used without any fine-tuning for GUI text. As shown in Table 5, FOTS achieves 45.52 % precision, 79.66 % recall and 57.93 % F1 score, which is significantly higher than Tesseract (29.4 % precision, 51.83 % recall and 37.31 % F1 score) and EAST (40.25 % precision, 72.03 % recall and 51.64 % F1 score). Both UI2CODE and REMAUI do some post-processing on Tesseract’s OCR results to filter out false positive results, but this still doesn’t significantly change the performance of GUI text detection. Since EAST and FOTS are specially designed for scene text recognition, their performance is significantly better than that of GUI text detection using a generic object detection model (see Table 5). EAST and FOTS can detect almost all text in GUI, including text on GUI components. At the same time, according to the previous definition in this paper, those texts on GUI components are considered as part of the component, rather than independent text components, which will affect the accuracy of EAST and FOTS in this experiment. Although these texts are incorrect results in this experiment, in the task of finally parsing into codes, the restoration of the entire GUI code must be guaranteed, so this paper uses the text results in these non-text components as one of its components Text property, to restore it in the final code.

This section draws the following conclusions for the research problem of text component detection and recognition performance analysis

- Text and non-text components of a GUI should be instrumented separately. In the experiments of detecting text components and non-text components separately and simultaneously, it is shown that the detection of the two components separately can achieve better overall detection performance.
- Using the deep learning scene text recognition model is better than OCR technology in the detection of GUI text components.

**Table 5:** Text component detection performance.

Method	Precision	Recall	F1-score
REMAUI	29.77	48.92	37.01
UI2CODE	27.26	48.15	34.81
EAST	40.25	72.03	51.64
OCR (Tesseract)	29.14	51.83	37.31
FOTS	45.52	79.66	57.93

### Experimental analysis conclusion

This section explores the application of the baseline model in GUI component detection tasks from two aspects: model performance, text detection and recognition. The application of general object detection methods to GUI component detection is analyzed, and three research questions are proposed. Through the analysis of the results of the exploratory experiments, these questions were answered, and it was concluded that the graphics processing method has a high component bounding box accuracy, and the deep learning object detection method has a high recall rate and can detect more components. Non-text components and text components should be detected separately, end-to-end text detection and recognition methods can efficiently detect text components and identify text within components as attributes of components. These conclusions provide important guiding significance for designing a new GUI code generation method based on component detection in the next section.

## Methodology

This section presents GUICG, a GUI code generation method that leverages component detection to enhance overall performance. We begin by introducing the architecture of GUICG, followed by an explanation of the image processing technique employed for GUI component detection. Subsequently, we describe the implementation details of the deep neural network model used for component detection. Furthermore, we design and implement an algorithm that integrates image processing and deep neural networks. Additionally, we present an end-to-end approach for text detection and recognition. Lastly, we discuss the method used to generate code based on the results of GUI component detection.

**Figure 2:**
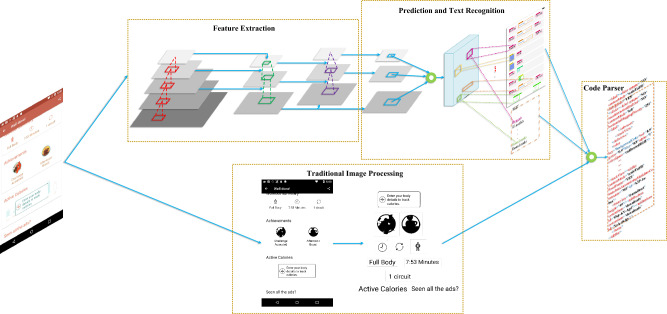
GUICG’s overall architecture, in which component detection combines traditional image processing methods and deep neural network methods.

### Overall architecture

The whole task is divided into object detection and text recognition and code generation. The architecture of *GUICG* is shown in Fig. 2. Input an image (GUI), run the image processing module, deep learning module, and text component detection and recognition module in parallel, and use the GUI elements detection algorithm that fuses deep neural network and traditional image processing technology to merge the detection results, and then integrate the results of the text detection and recognition module. Finally, use the parser to generate corresponding codes based on the detection results of the previous step. We use CSPDarkNet add SPP^37^. Then, the YOLO head uses the previously extracted features to make predictions, and the DenseNet performs text recognition on the bounding box area. In the GUI element detection algorithm based on traditional image processing techniques, we adopt the strategy from large blocks to small components to detect GUI object regions and then detect GUI elements. The GUI object detection model integrates deep neural network and traditional image processing technology in its architecture so that the results have a high recall rate and high detection accuracy. Finally, a code generator is used to generate code from the previous results.

### Image processing for GUI component detection

Image processing-based methods such as REMAUI^7^ and UI2CODE^34^ etc. detect bounding boxes that are usually accurate when locating GUI components. Therefore, this section designs an image processing method for GUI component detection to obtain component bounding boxes with high localization accuracy. Current dominant image processing methods use bottom-up strategies to gather fine details of objects (e.g., edges or contours) into objects. But this approach is vulnerable to complex background or GUI and objects in GUI components, the performance is poor.

**Figure 3:**
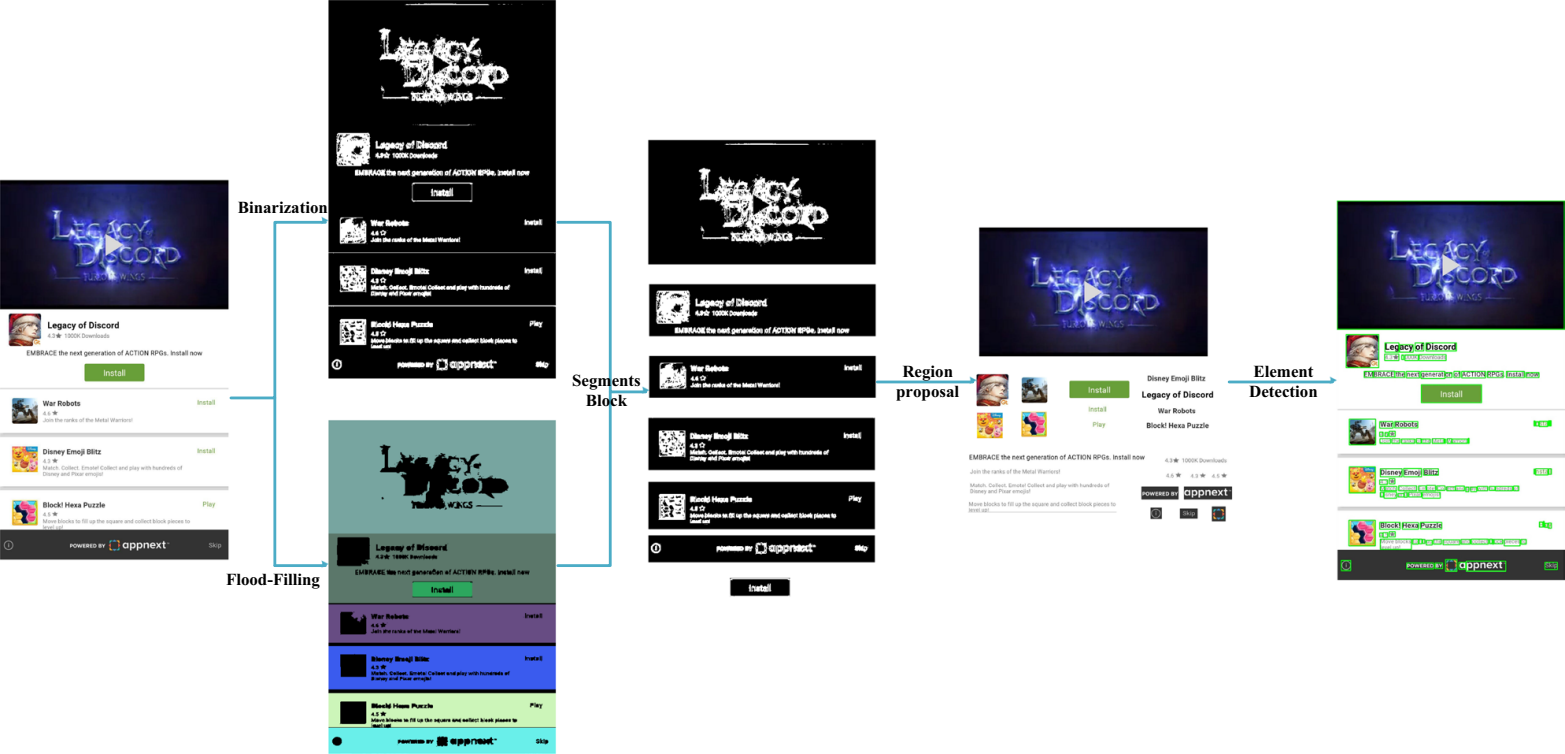
Image processing approach.

Therefore, this paper develops a novel image processing method (referred to as GUICG-IP), which uses a coarse-to-fine chunk-to-small component strategy to detect GUI component regions. Figure 3 shows the processing flow of GUICG-IP.

First, convert GUI image into a grayscale image, then use the flood filling algorithm^38^ on the grayscale image, and then use the shape recognition algorithm^39^ to determine whether these areas are rectangles. If is a rectangle then this area will be considered as a block. Then, Suzuki’s contour tracking algorithm^40^ is used to calculate the boundaries of the blocks and produce a block map. Next, our method generates a binary map of the input GUI image and segments out each detected block in the corresponding region of the binary map. Existing methods^6,7^ perform binarization by Canny edge detection^41^ and Sobel edge detection^42^, while these methods aim at preserving fine texture details in natural scene images. In this way, whatever content is displayed in the image is detected as an ImageView element. We therefore used a simple and effective binarization method^42^. The effect of binarization image processing is shown in Fig. 3, and the UI elements are easily recognized in the image. Finally, a connected component labeling algorithm^43^ is used to identify the GUI element regions in each binary block segment.

### Deep neural network for GUI component detection

The general-purpose deep learning target detection models Faster RCNN, FCOS, and YOLOv4 require enough training data, and different model designs require training data of different sizes to achieve stable performance. Moreover, unlike the loose definition of correct detection in object detection in natural scenes, detecting GUI components is a fine-grained recognition task that requires correct detection to cover the full area of GUI components as accurately as possible, while keeping the area of non-GUI components and other adjacent GUI components as small as possible. However, due to the characteristics of GUI components such as intra-class difference, inter-class similarity, dense arrangement and close distance between components, neither the anchor box-based model nor the anchor-free model can achieve this goal, that is, these based on Deep learning methods that generate component bounding boxes through statistical regression cannot meet the high localization accuracy requirements for GUI component detection.

Therefore, based on the YOLOv4 method, this section improves these unique features of GUI components, proposes CSPDarknet65 combined with the CSP^44^ network structure as the backbone network, and introduces the improved SPP^37^, The component detection algorithm of GUICG-OD uses CIoU^45^ as the loss function, the following are from various aspects of GUICG-OD The structure is described in detail.

#### CSPNet

CSPNet^44^ aims to solve the problem of increasing the amount of calculation caused by the repetition of gradient information in the network. Therefore, this paper redesigns part of the structure of the convolutional neural network, and adopts a cross-stage local structure on the basis of DarkNet. As shown in Fig. 5a, the cross-stage local structure divides the feature map output by the previous network into two parts through two 1 $\times$ 1 convolution kernels, one part is directly output without processing, and the other part is input into the original network perform calculations, and then concat the results of the two parts as the final output. After such processing, the network not only ensures that the feature map information is not lost, but also greatly reduces the amount of calculation.

#### CSPDarknet65

**Figure 4:**
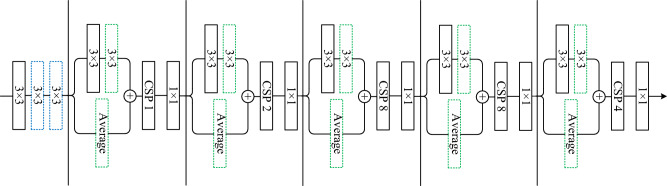
Illustration of backbone networks. Each rectangle includes Conv, BN and Mish. CSP N, N in {1, 2, 8, 4}, denoted as the residual block repeated N times with CSP structure. CSPDarknet65: additional residual block (blue block) and substituted downsampling residual block (green block).

The backbone network of GUICG-OD is CSPDarknet65, as shown in Fig. 4, which is based on the backbone network CSPDarknet53 structure and the backbone structure designed by Gao et al.^46^. CSPDarknet65 performs well in GUIS2code^2^.

#### Additional root block

**Figure 5:**
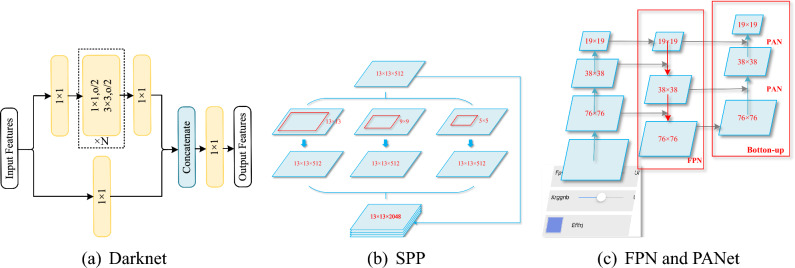
Architecture details. (**a**) is darknet structure, (**b**) is spatial pyramid pooling block, (**c**) is FPN and PANet in our model.

Performance can be improved by using a stack of 3 $\times$ 3 convolutional filters^47^. We increase the 3 $\times$ 3 convolution with stride 1 to three 3 $\times$ 3 convolutions, so that the network can obtain more local information in the image from a large number of inputs at the root stage, thereby extracting useful Features, as shown in the blue block in Fig. 5b.

#### Average pooling block

The Average Pooling block as a downsampling layer to speed up the gradient propagation in the network, as shown by the green block in Fig. 4. Compared with the downsampling block in CSPDarknet53, a 2$\times$2 average pooling layer with a stride of 2 is added. In front of the original 3$\times$3 convolutional layer, we add a 3$\times$3 convolutional layer with a stride of 1 to replace the downsampling layer. This structural improvement can avoid information loss during downsampling.

#### Spatial pyramid pooling

GUICG-OD uses a spatial pyramid pooling structure, which hardly reduces the network speed, but also significantly increases the network’s receptive field and improves the ability to extract contextual information. In this paper, the spatial pyramid pooling is improved, using four maximum pooling layers with a kernel size $k\times k$ , where $k = \{1, 5, 9, 13\}$, with a step size of 1 to process the feature map, and then cascading the four outputs to output, as shown in Fig. 5b.

#### Feature pyramid network

We aggregates feature maps extracted by backbone networks at different levels by adding a bottom-up feature pyramid structure after the FPN structure to provide a wide range of features for detectors at different levels^48^. The FPN structure contains bottom-up and top-down paths and the lateral connections between them. Since downsampling and upsampling affect the accuracy of object detection, top-down lateral connections between reconstruction layers and feature maps are required to better predict locations. However, in order to achieve better support for small target detection, this paper adds a bottom-up feature pyramid structure after the feature pyramid of the original structure, as shown in Fig. 5c. The bottom-up structure can transfer the rich positional information in the underlying feature map to the upper structure, and the top-down structure can transfer the rich semantic information in the upper feature map downward. Therefore, through this method, GUICG-OD The rich semantic features of GUI components and the location features of GUI components can be obtained at the same time, to improve the performance of GUI component detection and recognition.

#### Bounding box regression

We compared the Intersection over Union (IoU)^49^, the Generalized Intersection over Union (GIoU)^50^, the Distance Intersection over Union (DIoU)^45^, and the Complete Intersection over Union(CIoU)^45^. Finally, we use CIoU as the bounding box loss function. IoU is defined as

5 $$\begin{aligned} IoU = \frac{\mid A \bigcap B \mid }{ \mid A \bigcup B \mid } \end{aligned}$$

the CIoU loss function can be defined as

6 $$\begin{aligned} \mathscr{L}_{CIoU}=1-IoU+\frac{\rho ^{2} \left( \textbf{a},\textbf{b} \right) }{c^{2} } +\frac{ \upsilon }{ \left( 1-IoU \right) +\upsilon }\upsilon \end{aligned}$$

In summary, the backbone network of GUICG-OD is a CSPDarknet65 network with a cross-stage local structure and an additional root block and average pooling block. At the same time, an additional module of spatial pyramid pooling is added at the neck. After the feature pyramid, a bottom-up structure is added to make better use of the feature information extracted by the backbone network. Finally, the detection head directly uses the detection head of YOLOv3 and the bounding box loss function is changed to a more advanced CIoU.

### Fusion algorithms for GUI component detection

Input an image, run the image processing method and deep learning method in parallel, and get the extracted bounding boxes of the image processing and the deep learning method respectively. Finally, we use a fusion algorithm to get the final boxes. As shown in Algorithm 1, in the first step, the bounding box (i.e. *ipbox*) obtained by the image processing method is filtered, and when the IOU between *ipbox* and the bounding box (i.e. *odbox*) obtained by the deep learning method is greater than a certain threshold (e.g. 0.8), *ipbox* is kept, otherwise it is discarded, and *ipbox*1 is obtained in this way; in the second step, *odbox* is filtered, when the IOU between *odbox* and *ipbox*1 is greater than a certain threshold (e.g. 0.8), discard *odbox*, otherwise keep it and get *odbox*1. Afterwards, fix the position of *odbox*1 with the rule that each edge of *odbox*1 is moved to the nearest line, with the constraint that the distance moved cannot exceed a certain threshold (e.g. 10 pixels) and that the moved edge cannot cross the edge of *ipbox*1 to obtain the modified *odbox*2. Finally, the ensemble of *ipbox*1 and *odbox*2 is the final result.

**Algorithm 1:**
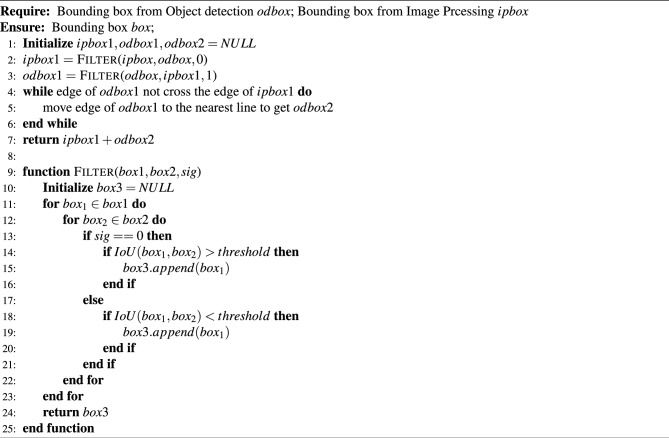
Fusion Algorithms.

### Text recognition

We consider the text regions of elements such as labels and text and choose FOTS^5^, the end-to-end text recognition method with the best text component detection performance in the exploratory experiment, as the network for text component detection and recognition. There are two kinds of text in the GUI image, one is the text component (such as TextView), and the other is the text in the component (such as the text in Button). FOTS can easily detect both text components as well as text belonging to GUI components. This paper considers the problem of identifying text in components. The text in these components should be considered as a part of the component. GUICG also recognizes it and takes these results as a text attribute of the component. In the final code generation task, these text attributes will be restored in the final code, which can ensure the degree of restoration of the entire GUI code.

### Code generation

The sequence of tokens generated from the previously mentioned network can then be compiled into the desired target language using traditional compilation methods. The algorithm for the code generator is shown in algorithm 2.

**Algorithm 2:**
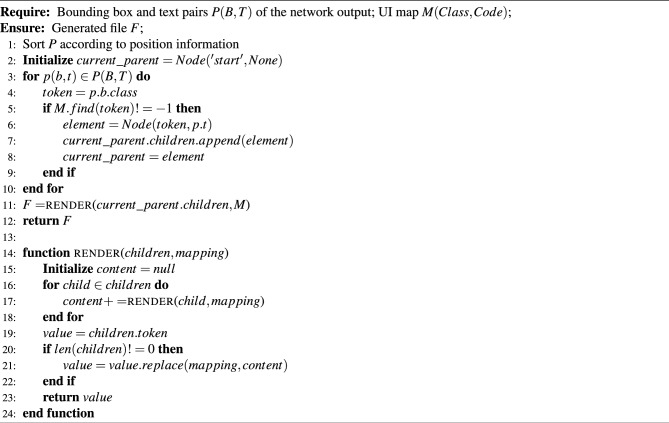
Code Generator.

The algorithm first organizes the result of the component detection part *P* into a tree data structure according to the location information. The attributes of *P* include the category of the component *class*, the bounding box of the component *b*, and the text of the component *t*. The transformation algorithm then completes the component-to-code conversion through a component matching graph *Map* containing category class-code pairs for each component. Use the depth-first search (DFS) method to traverse *P*, match the category of each component in *P* in *Map*, and then replace the position and text attributes of *code* of the successfully matched *Map* with the bounding box *b* and text *t* in *P*, and then write the result to the output file *F*, and so on until the entire result *P* is traversed and the code file *F* is output.

## Experiment

### Dataset and experiment setup

We use the Rico^33^ dataset to verify GUICG performance in experiments, and the information of the dataset is shown in Table 1. Fifteen common GUI elements in the Android platform are used in this paper. We split these 58,159 GUI images into training, validation, and test sets in a ratio of 8:1:1. Due to the accuracy requirement of GUI element detection, we set the IoU of the real box and the predicted box to 0.9 and 0.95 for the following study.

**Table 6:** Overall results of experiment (IoU > 0.9).

Method	Non-text elements			All elements		
	Precision	Recall	F1-score	Precision	Recall	F1-score
REMAUI^7^	0.151	0.205	0.173	0.296	0.449	0.357
YOLOv4^9^	0.395	0.486	0.436	0.397	0.488	0.438
GUIS2Code^2^	0.397	0.490	0.439	0.426	0.534	0.474
UIED^4^	0.431	0.469	0.449	0.490	0.557	0.521
GUICG (ours)	0.441	0.487	0.463	0.513	0.576	0.543

**Table 7:** Overall results of experiment (IoU > 0.95).

	Non-text elements			All elements		
Method	Precision	Recall	F1-score	Precision	Recall	F1-score
REMAUI^7^	0.098	0.134	0.113	0.185	0.347	0.241
YOLOv4^9^	0.264	0.459	0.335	0.287	0.391	0.331
GUIS2Code^2^	0.286	0.465	0.354	0.318	0.423	0.363
UIED^4^	0.379	0.388	0.383	0.401	0.455	0.426
GUICG (ours)	0.401	0.477	0.435	0.451	0.535	0.489

The GUICG is implemented in the PyTorch and was trained on four NVIDIA Tesla 16 GB GPUs with cuDNN acceleration. The operating system uses CentOS7.5, and the CPU is Intel(R) Xeon(R) E5-2640. The hyperparameters are set as follows: the number of training steps is 500,500; the step decay learning rate scheduling strategy is adopted, the initial learning rate is 0.01, and the 400,000th step and the 450,000th step are multiplied by a factor of 0.1; the momentum and weight decay are set to 0.9 and 0.0005, respectively.

For the evaluation of GUI element detection, we use precision, recall and F1 score to measure the performance of region detection.

### Contrast experiment

Tables 6 and 7 shows the overall GUI elements detection results, When IoU>0.9, among the four baseline models, GUIS2Code performed best for non-text elements in the deep learning-based approach (0.439 in F1), while UIED achieves the best F1 for non-text elements (0.449) and all elements (0.524) in the image-processing-based approach. But our model achieves much better F1 for both non-text elements (0.463) and all elements (0.543).

When IoU>0.95, all models experience some performance degradation, however, because our method integrates image processing methods, it has relatively strong element localisation accuracy and therefore still has relatively strong performance even with a strict threshold. Our method only degrades 0.062 in accuracy, compared to 0.089 for UIED and 0.1-0.2 for all other methods. In this experiment, GUICG achieves a more significant advantage, achieving an F1 score of 0.489 on all elements, which exceeds the F1 score of the best of the other methods, UIED(0.426), by 6.3%.

### Ablation

The results of the ablation experiment are shown in the Tables 8 and 9. GUICG-IP denotes Image Processing for GUI element detection, GUICG-OD denotes object detection for GUI element Detection.

As shown in Table 8, compared with the baseline model YOLOv4, the improved GUICG-OD based on it exceeds YOLOv4 in three indicators: precision (0.435), recall (0.562) and F1 score (0.490). In the two IoU threshold experiments, GUICG-IP achieved higher accuracy than GUICG-OD, which further illustrates the advantages of image processing methods in the accuracy of GUI component detection bounding box prediction. However, its recall rate is relatively lower, further illustrating that deep learning-based object detection methods are able to detect more components in GUI images in comparison. Finally, GUICG, which integrates the two methods, maintains the advantages of the two methods in both precision (0.513) and recall (0.576), and achieves better results than the single method in the F1 score (0.543).

**Table 8:** Ablation experiment (IoU > 0.9).

Method	Precision	Recall	F1-score
GUIS2Code^2^	0.426	0.534	0.474
GUICG-IP	0.505	0.413	0.454
GUICG-OD	0.435	0.562	0.490
GUICG	0.513	0.576	0.543

**Table 9:** Ablation experiment (IoU > 0.95).

Method	Precision	Recall	F1-score
GUIS2Code^2^	0.318	0.423	0.362
GUICG-IP	0.453	0.308	0.367
GUICG-OD	0.334	0.460	0.387
GUICG	0.451	0.535	0.489

Table 10 shows the region classification results for GUICG and three deep learning baselines. We can see that our method outputs more regions of true-positive GUI elements and achieves higher classification accuracy (0.87 for non-text elements and 0.94 for all elements) than the other three deep models.

**Table 10:** Region classification results for TP regions.

Method	Non-text elements		All elements	
	#bbox	Accuracy	#bbox	Accuracy
YOLOv4^9^	16085	0.69	32245	0.69
GUIS2Code^2^	17129	0.72	34759	0.73
UIED^4^	21977	0.85	52458	0.91
GUICG (ours)	22375	0.87	54289	0.94

## Discussion

The method GUICG in this paper has achieved good results on the Rico dataset, and the GUI generated by the generated code is basically the same as the original image. However, the effect of GUICG is still extremely limited. The F1 score in component detection is only 0.543, and it can only recognize some components in the GUI image. Therefore, in the future research ideas of this topic, it can be expanded from the following points:

- Current Rico datasets based on real applications only contain GUI images of Android applications, lacking real datasets for iOS and web interfaces. Current datasets including iOS and web interfaces such as pix2code are manually created images, and the generated interfaces are based on manually set rules, which are quite different from the interfaces in real applications. In follow-up research, screenshots of websites and their associated HTML code or iOS apps can be grabbed to create datasets.
- The current neural network-based method needs to rely on DSL files to describe GUI images, resulting in a lot of work for the creation of data sets. In the future, we can consider how to abandon the dependence of DSL to improve the efficiency of GUI automatic code generation methods.

## Conclusion

In this paper, we propose GUICG, a GUI code generator that combines deep neural networks and image processing. The proposed method is based on a novel fusion of deep neural networks and image processing techniques, and achieves state-of-the-art performance in detecting GUI elements from GUI images. It takes GUI images as input and generates interface codes suitable for various platforms. Our fusion algorithm improves the recognition rate of GUI components. Furthermore, we consider the detection and recognition of text components. By employing an end-to-end text recognition method, we can simultaneously recognize text in non-text components and utilize the text information for the generated code. Our empirical evaluation demonstrates that GUICG outperforms current state-of-the-art methods across multiple evaluation metrics, achieving an impressive F1 score of 0.543. Overall, our novel GUICG model excels in both component detection and code generation.

## Data Availability

Correspondence and requests for data and materials should be addressed to B.C.

## Additional information

Reprints and permissions information is available at www.nature.com/reprints.
